# Low Rates of Mother-to-Child HIV Transmission in a Routine Programmatic Setting in Lilongwe, Malawi

**DOI:** 10.1371/journal.pone.0064979

**Published:** 2013-05-31

**Authors:** Maria H. Kim, Saeed Ahmed, Geoffrey A. Preidis, Elaine J. Abrams, Mina C. Hosseinipour, Thomas P. Giordano, Elizabeth Y. Chiao, Mary E. Paul, Avni Bhalakia, Debora Nanthuru, Peter N. Kazembe

**Affiliations:** 1 Baylor College of Medicine International Pediatric AIDS Initiative at Texas Children’s Hospital, Baylor College of Medicine, Houston, Texas, United States of America; 2 International Center for AIDS Care and Treatment Programs, Mailman School of Public Health, Columbia University, New York New York, United States of America; 3 University of North Carolina Project, Lilongwe, Malawi; 4 University of North Carolina School of Medicine, Chapel Hill, North Carolina, United States of America; 5 Department of Medicine, Baylor College of Medicine, Houston, Texas, United States of America; 6 Baylor College of Medicine-Abbott Fund Children’s Clinical Center of Excellence, Lilongwe, Malawi; University of North Carolina School of Medicine, United States of America

## Abstract

**Background:**

The *Tingathe* program utilizes community health workers to improve prevention of mother-to-child transmission (PMTCT) service delivery. We evaluated the impact of antiretroviral (ARV) regimen and maternal CD4+ count on HIV transmission within the *Tingathe* program in Lilongwe, Malawi.

**Methods:**

We reviewed clinical records of 1088 mother-infant pairs enrolled from March 2009 to March 2011 who completed follow-up to first DNA PCR. Eligibility for antiretroviral treatment (ART) was determined by CD4+ cell count (CD4+) for women not yet on ART. ART-eligible women initiated stavudine-lamivudine-nevirapine. Early ART was defined as ART for ≥14 weeks prior to delivery. For women ineligible for ART, optimal ARV prophylaxis was maternal AZT ≥6 weeks+sdNVP, and infant sdNVP+AZT for 1 week. HIV transmission rates were determined for ARV regimens, and factors associated with vertical transmission were identified using bivariate logistic regression.

**Results:**

Transmission rate at first PCR was 4.1%. Pairs receiving suboptimal ARV prophylaxis were more likely to transmit HIV (10.3%, 95% CI, 5.5–18.1%). ART was associated with reduced transmission (1.4%, 95% CI, 0.6–3.0%), with early ART associated with decreased transmission (no transmission), compared to all other treatment groups (p = 0.001). No association was detected between transmission and CD4+ categories (p = 0.337), trimester of pregnancy at enrollment (p = 0.100), or maternal age (p = 0.164).

**Conclusion:**

Low rates of MTCT of HIV are possible in resource-constrained settings under routine programmatic conditions. No transmissions were observed among women on ART for more than 14 weeks prior to delivery.

## Background

Effective medical interventions for prevention of mother-to-child transmission of HIV (PMTCT) have been known since the early 1990s, and in developed countries, new pediatric HIV infections have become increasingly rare [1], [2]. Globally, in resource-limited settings, studies have demonstrated the efficacy or effectiveness of various PMTCT interventions, including single-dose nevirapine (sdNVP), combination prophylaxis, maternal antiretroviral treatment (ART), and extended infant prophylaxis [3]–[6]. These studies have informed the development of World Health Organization (WHO) guidelines with simple and effective interventions that can result in transmission rates of less than 5% feasible, even in breastfeeding populations [7], [8].

Despite these advances, an estimated 330,000 new infections occur in children every year, the vast majority attributed to vertical transmission [9]. Persistent poor outcomes in developing countries generally are described as a result of HIV-infected mothers and exposed infants not receiving medical services [10], [11]. However, outside the unique environment of controlled research studies, few reports [12]–[14] have documented the real-world effectiveness of PMTCT interventions when properly administered within routine programmatic settings.

In partnership with the Malawi Ministry of Health, the Baylor College of Medicine *Tingathe* Program (meaning “yes we can” in the local Chichewa language) utilizes community health workers (CHWs) to improve uptake and delivery of routine, available PMTCT interventions [14]. *Tingathe* CHWs support women to engage in longitudinal care throughout the full PMTCT cascade, starting with diagnosis of the woman at antenatal care (ANC) and ending with final diagnosis of the infant. This report documents transmission outcomes at first PCR for mother-infants pairs enrolled in the *Tingathe* PMTCT program in Lilongwe, Malawi.

## Methods

### Ethics Statement

This retrospective study of routinely collected programmatic data was performed in full accordance with the guidelines for research outlined by the National Health Sciences Research Committee of Malawi and the Baylor College of Medicine institutional review board. All data collected were part of the delivery of routine program services. For this study, both the Malawi and Baylor review boards waived the need for written consent. Verbal consent for HIV testing was obtained by nationally certified HIV counselors in accordance with Malawi National HIV Counseling and Testing guidelines. Data were de-identified prior to analysis.

### Study Design

We conducted a retrospective cohort analysis of mother-infant pairs enrolled in the *Tingathe* program, March 2009 to March 2011, who had a first DNA PCR result available by January 28, 2012. Our primary outcome was infant HIV infection at first DNA PCR. PCR was routinely performed at the first postnatal visit, as early as 6 weeks of age.

### Intervention Setting and Patient Population

The *Tingathe* PMTCT pilot program took place in Area 25 and Kawale, two large peri-urban communities in Lilongwe, Malawi. The estimated population is 310,000 people, with 15,000 deliveries per year, 2000 HIV-exposed infants delivered per year, and adult HIV prevalence of 12% [15]. Over 96% of pregnant women attend at least one antenatal visit [16], and 99% of antenatal clinic (ANC) attendees were tested for HIV [11], [17]. HIV testing at antenatal care was performed via opt-out testing as per Malawi Ministry of Health HIV guidelines [18].

### The *Tingathe* Program

The *Tingathe* PMTCT program has been described in detail elsewhere [14]. In brief, one *Tingathe* community health worker (CHW) is assigned to each HIV-infected pregnant woman upon diagnosis or enrollment into antenatal care (ANC). The CHW supports the woman to engage in longitudinal care throughout the full PMTCT cascade, starting with her diagnosis at ANC, and including CD4+ cell count (CD4+), delivery of CD4+ results, enrollment into ART clinic and initiation of ART if eligible, delivery of antiretroviral (ARV) prophylaxis to mother and infants, and DNA PCR testing of the infant. CHWs follow their clients at their homes and health centers, from initial diagnosis until confirmation of definitive HIV-uninfected status after cessation of breastfeeding or successful ART initiation for HIV-infected infants. Receipt of ARV regimen is recorded only upon confirmation with the mother after delivery to verify that medication has actually been ingested, not just dispensed [19]. HIV-infected women identified at labor and delivery or after the birth of the infant are also followed and provided services but were not included in this analysis.

### Routine PMTCT Services and ARV Regimens Available at Intervention Sites

All PMTCT clinical care was provided in accordance with Malawi Ministry of Health and WHO guidelines [7], [18]. In brief, all women not already on ART at the time of enrollment had a venipuncture performed to obtain blood for CD4+ cell count. If a woman met ART eligibility criterion determined by CD4+, she received the fixed-dose combination ART regimen of stavudine (d4T)+lamivudine (3TC)+nevirapine (NVP). In August 2009, CD4+ criterion for ART eligibility in HIV-infected pregnant women in Malawi changed from CD4+ ≤250 cells/mm^3^ to CD4+ ≤350 cells/mm [3], [20]. Those women who did not meet eligibility criterion received combination ARV prophylaxis with zidovudine (AZT) at 28 weeks, sdNVP at the onset of labor, and a 7-day AZT/3TC tail. Infants received sdNVP and AZT for 1 week postnatally if their mothers received AZT for greater than 4 weeks prior to delivery, and for 4 weeks postnatally if their mothers received AZT for less than the minimum 4 weeks prior to delivery.

### ARV Regimens

**Figure 1:**
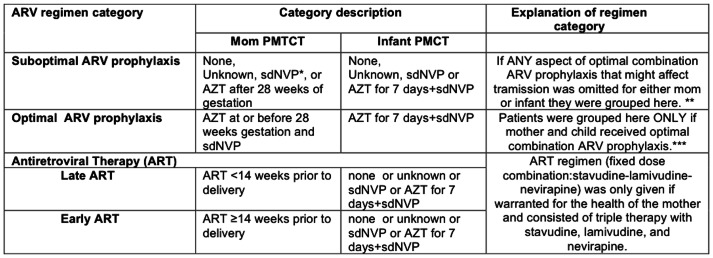
Description and explanations of antiretroviral regimens used for analysis. *sdNVP: single dose of nevirapine for the mother taken at the onset of labor and a single dose of nevirapine for the infant administered within 72 hours of delivery. **Due to low patient numbers in each individual grouping, these suboptimal interventions were grouped together. ***Based on 2006 WHO PMTCT guidelines. Abbreviations: ARV (antiretroviral), ART (antiretroviral therapy), AZT (zidovudine).

Figure 1 details the ARV regimens used for this study. Optimal ARV prophylaxis was defined as the mother-infant pair receiving complete ARV prophylaxis (including AZT, sdNVP) at the correct time with the correct doses. Suboptimal ARV prophylaxis included mother-infants who received no prophylaxis or sdNVP only or AZT only. Due to the low numbers in each group, all these suboptimal interventions were grouped together. Therefore, the suboptimal ARV prophylaxis group included all mother-infant pairs for which any component of the optimal ARV prophylaxis regimen was either omitted or given incorrectly. Mother-infant pairs in which the women missed only the post-delivery AZT/3TC tail were included in the optimal ARV prophylaxis group, as the tail is provided to prevent resistance in the mother and is not thought to influence transmission [20], [21]. All women who received ART for their own health were grouped in the ART group, with those who initiated ART at least 14 weeks prior to delivery further categorized as early ART.

### Infant HIV Testing

In accordance with national guidelines, infants received their first HIV test by DNA PCR by Dried Blood Spot (DBS) at or after 6 weeks of life [18]. Transmission was defined as positive PCR result at first DNA PCR.

### Statistical Analysis

Electronic clinical records of all HIV-infected pregnant women enrolled March 2009 -March 2011 were reviewed. Data were de-identified prior to analysis. Aggregate data were reported as mean with standard deviation (SD) or median with interquartile range (IQR) based on normality. We computed HIV transmission rates as proportions with 95% confidence intervals (CI), using the modified Wald method. Proportions were compared initially with a univariate global Pearson chi-square test. When global testing indicated a significant difference somewhere within the data, bivariate pair-wise, *post hoc* comparisons were made using either chi-square or Fisher exact tests, with alpha adjusted for multiple comparisons using Bonferroni's correction. Only pair-wise comparisons with the p-value less than adjusted alpha were considered significant. Continuous variables were compared between two groups using an independent-samples t test. All statistical analyses were performed using IBM SPSS Statistics (version 20.0.0, IBM Corporation, Armonk, NY).

## Results

**Figure 2:**
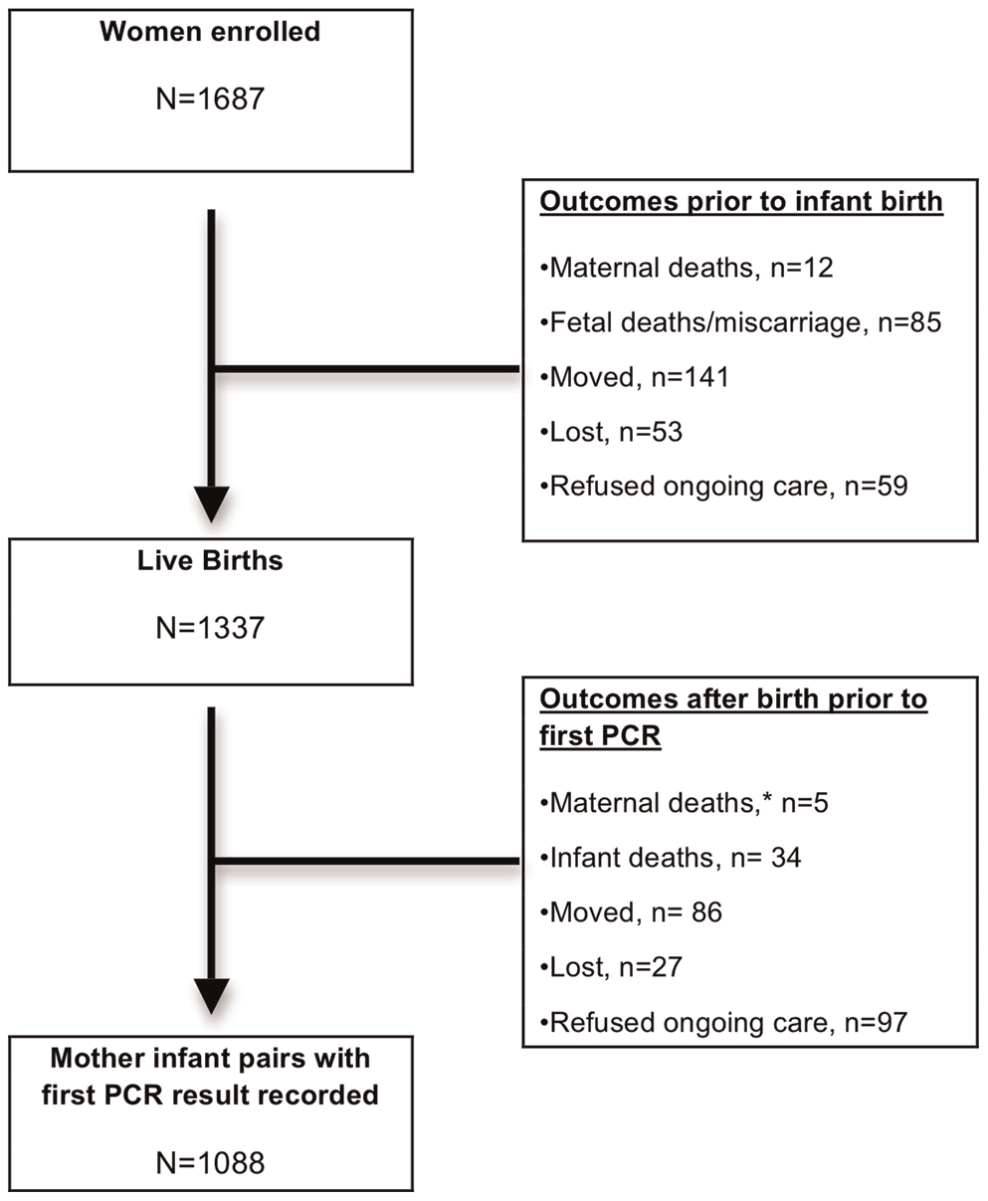
Outcomes for HIV-infected pregnant women and HIV-exposed infants enrolled in the *Tingathe* program. After the maternal deaths, all five infants were transferred to the care of another caregiver and moved out of the program catchment area.

From March 2009 to March 2011, 1687 HIV-infected pregnant women were enrolled into the *Tingathe* PMTCT program. Figure 2 details the outcomes for these women and their exposed infants through the first infant DNA PCR. There were 97 maternal-fetal-infant deaths as well as 253 who moved from the catchment area, were lost or refused ongoing care prior to infant birth. Of 1337 recorded live births, there were 39 maternal-infant deaths as well as 210 who either moved, were lost, or refused ongoing care. Therefore, there were 1088 (81.4%) with a first PCR result recorded.

### Baseline Characteristics

Table 1 describes baseline characteristics of women in our cohort. Of the 1088 mother-infant pairs with first PCR result recorded, the mean age of the woman was 27.8 years (SD = 5.2). Median gestational age at program entry was 24 weeks (IQR 20–28), and 699 (64.2%) were in their first or second trimesters. The majority of women (71.7%) were newly diagnosed with HIV infection at program entry. Of those newly diagnosed, 307 (28.2%) were eligible for ART. Of the 307 women eligible for ART, 192 (62.5%) were eligible using the threshold of ≤250 CD4+ cell/mm^3^ and 115 (37.5%) were eligible using the ≤350 cell/mm^3^ threshold. There were 262 (24.1%) women already on ART at program entry. Most women (65.1%) had not disclosed their HIV status to their partners. Average baseline CD4+ cell count was 414 cells/mm^3^ (SD = 227) for the 803 women not on ART at the time of entry into the program and for whom CD4+ was available.

**Table 1:** Characteristics of 1088 HIV positive pregnant women at enrollment in the Tingathe program.

Characteristic		Result
**Age, mean years (SD)**		27.8 (5.2)
**Trimester of pregnancy: n(%)**		
	First	54 (5.0)
	Second	645 (59.3)
	Third	384 (35.3)
	Missing data	5 (0.4)
**HIV status at enrollment, n (%)**		
	Newly diagnosed as HIV-infected	780 (71.7)
	Already known to be HIV-infected	308 (28.3)
**ART eligibility at enrollment**		
	On ART	262 (24.1)
	Eligible but not on ART	307 (28.2)
	Not Eligible	496 (45.6)
	Missing data	23 (2.1)
**CD4+ cells/mm^3^ at PMTCT entry for women not already on ART, n/N (%)** a		
	≤ 250	192 (17.6)
	251–349	149 (13.7)
	350–499	224 (20.6)
	≥500	238 (21.8)
	Missing data	23 (2.1)
**Partner Disclosure status at enrollment, n (%)** b		
	Partner involved and disclosed	319 (29.3)
	Partner involved, not disclosed	708 (65.1)
	Partner not involved	61 (5.6)

a Of the 1088 mother-infant pairs with first DNA PCR results, 262 mothers were already on ART, 23 with no corresponding maternal CD4 results, therefore 803 available CD4 results with corresponding PCR results.

b Partner disclosed defined as partner having knowledge of maternal HIV status. Partner non-involved defined as a partner who has died, or is otherwise separated from the mother.

Abbreviations: ART (antiretroviral therapy), CD4+ (CD4+ cell count), PMTCT (prevention of mother-to-child transmission).

Compared to women whose infants received a first PCR, those who were lost to follow-up (lost, moved, and refused to participate) were younger (mean maternal age 26.9 [SD 5.8] versus 27.8 [SD 5.2] years, p = 0.002), greater proportion enrolled during the first trimester of pregnancy [9% vs. 5% in 1^st^ Trimester; p = 0.001]; greater proportion newly diagnosed with HIV at enrollment [84.8% vs. 71.7%; p<0.001]; greater proportion on ART at enrollment [24.6% vs. 13.2%; p<0.001]; and greater proportion with partners who were not aware of the mother’s HIV status [29.3% vs. 17.2%; p<0.001]. There were no differences noted in baseline CD4 cell count.

Among the 1088 infants, median age at first PCR was 1.7 (IQR 1.5–2.5) months: 96.6% were tested at <6 months of age and 81% were tested at ≤ 12 weeks of age.

### HIV Transmission and ARV Regimens

Of the 882 (81.1%) performed at ≤ 12 weeks, 38 (4.3%) were positive; of the 104 (9.6%) performed at >12–16 weeks, 3 (2.9%) were positive; of the 65 (6%) performed at >16–24 weeks, 3 (4.6%) were positive; of the remaining 37 (3.4%) performed, 1 (2.7%) was positive. There was no difference between the transmission rates of those with first PCR performed at ≤ 12 weeks of age versus those >12 weeks of age (4.3% versus 3.4%; p = 0.555).

Among the 485 women who received ART, 187 (38.6%) received ART late (ART <14 weeks prior to delivery). Median duration of antenatal ART was 19 (IQR 8.9–104.9) weeks for the ART group as a whole, 82 (IQR 22–159) weeks for the early ART group, and 7.1 (IQR 4.4–9.9) weeks for the late ART group. Of the women who received early ART 194/298 (65%) were on ART at least 40 weeks prior to infant birth.

**Figure 3:**
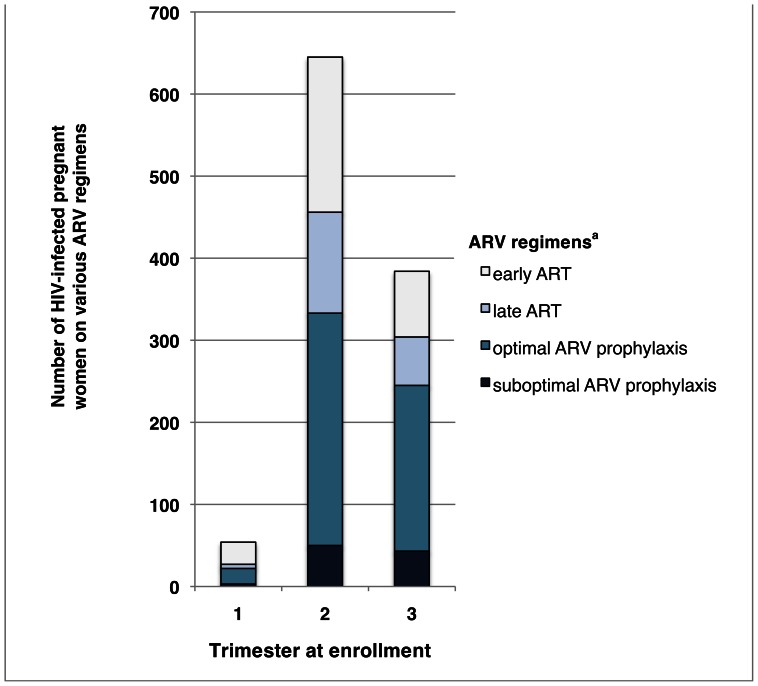
Antiretroviral regimen by trimester at enrollment. ^a^For explanations of ARV regimen categories please refer to Figure 1. Abbreviations: ART (antiretroviral therapy), ARV (antiretroviral).

Figure 3 illustrates ARV regimen by trimester. Of those women who presented in the 1^st^ trimester, 85% received either early ART (50%) or optimal prophylaxis (35%). Of those who presented in the 2^nd^ trimester, 73% received either early ART (29%) or optimal prophylaxis (44%). Finally, of those women who presented in the 3^rd^ trimester, 73% received either early ART (20%) or optimal prophylaxis (53%).

Table 2 documents how HIV transmission rates varied according to ARV regimen received by the mother-infant pair. Those who received suboptimal ARV prophylaxis had the highest rate of transmission (10.3%, 95% CI, 5.5–18.1%). Those who received ART had reduced transmission (1.4%, 95% CI, 0.6–3.0%), whereas early ART (initiated ART ≥14weeks prior to delivery) was associated with the lowest transmission, with no transmissions among these 298 women (p<0.001 compared to all other ARV regimens; Table 2).

**Table 2:** Vertical HIV transmission rates at first PCR by antiretroviral regimen.

ARV regimen category		First PCR positiveN = 45	First PCR negativeN = 1043	Vertical HIV transmission rate at first PCR^b^	p-value^c^
**ARV regimen category** a **, n (%)**					<0.001
	Prophylaxis	38 (84.4)	565 (54.2)	6.3 (4.6–8.6)	
	Antiretroviral therapy	7 (15.6)	478 45.8)	1.4 (0.6–3.0)	
**ARV regimen category** a **, n (%)**					**<0.001**
	Suboptimal ARV prophylaxis	10 (22.2)	86 (8.3)	10.3 (5.5–18.1)	0.001^c^
	Optimal combination ARV prophylaxis	28 (62.2)	479 (45.9)	5.5 (3.8–7.9)	0.032
	Late ART (initiated <14 weeks prior to delivery)	7 (15.6)	180 (17.3)	3.7 (1.7–7.7)	0.767
	Early ART (initiated ≥14 weeks prior to delivery)	0 (0.0)	298 (28.5)	0.0 (0.0–1.5)	0.001^c^

a For explanations of ARV regimen categories please refer to Figure 1.

b Early vertical transmission rate calculation: number positive first PCR results/number of total first PCR results.

c Denotes p< alpha, adjusted to 0.0125 to account for multiple comparisons. *Post hoc* analysis performed only if the global p-value (in bold) was <0.05. (see Methods).

Abbreviations: PCR (polymerase chain reaction)- DNA PCR test for HIV, ART (antiretroviral therapy),

### Factors Associated with HIV Transmission

Factors significantly associated with HIV transmission on bivariate analysis included ART eligibility, type of ARV regimen, and the duration of antenatal ART (Table 3). Specifically, women already receiving ART at the time of enrollment or women initiated on ART early in the course of the pregnancy were less likely to transmit HIV. Receiving ART for at least 14 weeks antenatally (early ART) was associated with decreased transmission (no transmission) compared to all other forms of ARV prophylaxis (p = 0.001), whereas receiving ART for less than 14 weeks (late ART) was associated with increased transmission of HIV (p = 0.001).

**Table 3:** Characteristics of women and infants associated with positive first PCR versus negative first PCR.

Characteristic		Infants with first PCR positive(N = 45)	Infants with first PCR negative(N = 1043)	p-value
**Maternal Age, mean years (SD)**		26.7 (4.9)	27.9 (5.3)	0.164
**Trimester of pregnancy: n/N (%)**				0.100
	First	5/45 (11.1)	49/1038 (4.7)	
	Second	28/45 (62.2)	617/1038 (59.4)	
	Third	12/45 (26.7)	372/1038 (35.8)	
**HIV status at enrollment, n/N (%)**				0.355
	Newly diagnosed as HIV-infected	35/45 (77.8)	745/1043 (71.4)	
	Already known to be HIV-infected	10/45 (22.2)	298/1043 (28.6)	
**ART eligibility at enrolment, n/N (%)**				**0.015**
	On ART	2/39 (5.1)	260/1026 (25.3)	0.002^a^
	Eligible but not on ART	15/39 (38.5)	292/1026 (28.5)	0.176
	Not Eligible	22/39 (56.4)	474/1026 (46.2)	0.210
**CD4+ cells/mm^3^ at PMTCT entry for women not already** **on ART, n/N (%)** b				0.337
	≤ 250	8/37 (21.6)	184/766 (24.0)	
	251–349	10/37 (27.0)	139/766 (18.1)	
	350–499	12/37 (32.4)	212/766 (27.7)	
	≥500	7/37 (18.9)	231/766 (30.2)	
**ARV regimen category, n/N (%)** c				<0.001
	Prophylaxis	38 (84.4)	565 (54.2)	
	Antiretroviral therapy	7 (15.6)	478 45.8)	
**ARV regimen category, n/N (%)** c				**<0.001**
	Suboptimal ARV prophylaxis	10/45 (22.2)	86/1043 (8.2)	0.001^a^
	Optimal comb. ARV prophylaxis	28/45 (62.2)	479/1043 (45.9)	0.032
	Antiretroviral therapy	7/45 (15.6)	478/1043 (45.8)	<0.001^a^
**Duration of antenatal ART, n/N (%)**				0.001^a^
	Late ART (initiated <14 weeks prior to delivery)	7/7 (100.0)	180/478 (37.7)	
	Early ART (initiated ≥14 weeks prior to delivery)	0/7 (0.0)	298/478 (62.3)	
**Partner Disclosure status at enrollment, n/N (%)** d				0.235
	Partner involved and disclosed	13/45 (28.9)	306/1043 (29.3)	
	Partner involved, not disclosed	32/45 (71.1)	676/1043 (64.8)	
	Partner not involved	0/45 (0.0)	61/1043 (5.8)	
**Delivery Location, n/N (%)**				0.293
	Home	2/45 (4.4)	24/1043 (2.3)	
	Health center	43/45 (95.6)	1017/1043 (97.5)	
	Traditional Birth Attendant	0/0 (0.0)	2/1043 (0.2)	
**Feeding Choice, n/N (%)**				0.708
	Breast feeding	44/44 (100.0)	1020/1036 (98.5)	
	Mixed	0/44 (0.0)	1/1036 (0.1)	
	Replacement feeding	0/44 (0.0)	15/1036 (1.4)	

a Denotes p< alpha, equal to 0.05 for 2-category comparisons or adjusted to 0.017 for 3-category comparisons. *Post hoc* analysis performed only if the global p-value (in bold) was <0.05. (see Methods).

b 1088 available PCR results, of these 262 mothers were already on ART, 23 with no corresponding maternal CD4 results, therefore 803 available CD4 results with corresponding PCR results.

c For explanations of ARV regimen categories please refer to Figure 1.

d Partner disclosed defined as partner having knowledge of maternal HIV status. Partner non-involved defined as a partner who has died, or is otherwise separated from the mother.

Abbreviations: PCR (polymerase chain reaction)- DNA PCR test for HIV, ART (antiretroviral therapy), ARV (antiretroviral), CD4+ (CD4+ cell count), PMTCT (prevention of mother-to-child transmission).

### HIV Transmission by Baseline CD4+ Cell Count

CD4+ counts were only obtained on women not on ART at the time of program initiation. Of these 826 women, 803 (97.2%) had CD4+ counts recorded at the time of program registration. No difference in vertical transmission rate was detected according to CD4+ level at enrollment (p = 0.302).

Maternal age, gestational trimester, HIV status and CD4+ count at the time of enrollment, partner disclosure, delivery location, and breast-feeding status were also not associated with HIV transmission. (Table 3).

### Recommended ARV Regimen not Received

Of the 1088 mother-infant pairs, 96 (8.8%) received suboptimal ARV prophylaxis. Even among the 507 women who received optimal combination ARV prophylaxis, 34 (6.7%) did not receive the recommended one-week AZT/3TC tail. Therefore, 130 (12%) did not receive the recommended regimen. Furthermore, 31 of 307 ART eligible women did not initiate ART even after infant delivery.

## Discussion

Results from our cohort of HIV-infected women and their exposed infants suggest that low rates of vertical transmission of HIV are possible in resource-constrained settings. We observed an overall transmission rate at first DNA PCR of 4.1%. These results compare favorably with other reports from resource-constrained settings, including studies limited to women on ART [13], [22], [23] and PMTCT clinical trials [5], [6], [20].

Study results have been mixed regarding the relationship between immunosuppression and vertical transmission [1], [2], [13], [24], [25]. In the context of appropriate PMTCT delivery that includes ART for eligible women, our results suggest that CD4+ cell count at the time of entry into PMTCT does not significantly impact transmission. In our cohort, those women at highest risk of transmitting the virus due to severe immunosuppression (<250 cells/mm^3^) had transmission rates that were not significantly different from those with less immunosuppression.

As expected, ART was more protective than was any other ARV regimen. Furthermore, starting ART at least 14 weeks prior to delivery (early ART) yielded the most benefit in reducing transmission, with no transmissions noted in the early ART group. These results are consistent with findings that complete viral suppression usually takes place after 12 to 16 weeks of therapy [24], [26], [27]. Other clinical studies have reported similar results, including a study from Zambia that found that women who received ART for 4 or fewer weeks had a 5.5- fold increased odds of transmission when compared with women on HAART for at least 13 weeks [22]. Similarly, all women enrolled in the Dream Program in Malawi, Tanzania, and Mozambique were started on ART at 25 weeks and were found to have a transmission rate of just 0.8–1.2% at 1 month [23]. Importantly, over one third of the women in our cohort who were on ART received it for fewer than 14 weeks prior to delivery (i.e., less than the optimal longer duration). Moreover, over one-third of our cohort was enrolled during the third trimester. Therefore, our results not only confirm that early initiation of ART yields maximal benefits in reducing transmission but also highlight that increasing efforts need to be made to ensure that women present to antenatal care sooner and early initiation of ART be a priority PMTCT intervention.

Although the patients in this cohort were all treated under the 2006 WHO guidelines [7], there are some implications for policy makers as they implement the 2010 WHO guidelines (Table 4) [28]. There is similar complexity and steps between the combination prophylaxis of 2006 and both option A and option B from 2010. Most important, as with the WHO 2006 guidelines, the WHO 2010 guidelines still require measurement of CD4+ to determine which regimen to administer for option A and to determine whether ART should be stopped after breastfeeding for option B. This complexity can lead to errors made by both the healthcare system and patients. In our program, even with CHW case management, 12% of patients did not receive the complete recommended regimens. In settings where CHW case management is not present, the risk of errors is likely to be even higher. It appears that the multiple steps necessary for options A and B may be challenging for patients to negotiate.

**Table 4:** Descriptions of WHO Option A, B, and B+.

WHO option	Woman-Treatment(if CD4≤350)	Woman-Prophylaxis(if CD4>350)	Breastfeeding Infant
**WHO option A** *Requires CD4*	Triple ART	AZT from 14 weeks’ gestation, sdNVP andAZT/3TC at onset of labor, and AZT/3TCfor 7 days postpartum	Daily NVP from birth to 1 week after all exposure to breast milk has ended
**WHO option B** *Requires CD4*	Triple ART	Triple ARV from 14 weeks’ gestation until 1 weekafter all exposure to breast milk has ended	Daily NVP from birth to 6 weeks
**WHO option B+** *CD4 not required*	Triple ART *for life*	Triple ART *for life*	Daily NVP from birth to 6 weeks

ƒƒƒÏ.

Abbreviations: AZT (zidovudine), ART (antiretroviral therapy), ARV (antiretroviral); sdNVP (single dose nevirapine), NVP (nevirapine).

World Health Organization (2012) Programmatic Update. Use of Antiretroviral Drugs for Treating Pregnant Women and Preventing HIV Infection in Infants. Executive Summary. Geneva, Switzerland: World Health Organization.

Schouten EJ, Jahn A, Midiani D, Makombe SD, Mnthambala A, et al. (2011) Prevention of mother-to-child transmission of HIV and the health-related Millennium Development Goals: time for a public health approach. Lancet 378∶282–284.

The Malawi Ministry of Health recently embarked on a novel PMTCT strategy called option B+ (Table 4) that starts all HIV-infected pregnant women on triple ARV prophylaxis regardless of eligibility for ART and, therefore, requires no CD4+ testing [29], [30]. Our results support this approach for several reasons. First, given the high rate of errors with combination prophylaxis and ART threshold initiation in our cohort, option B+ may provide a simpler alternative because all HIV-infected pregnant women will be receiving the same triple ARV for life without the need to first assess CD4+ cell counts. Second, the multiple challenges to accessing reliable CD4+ testing in resource-constrained settings renders a system that obviates the need for CD4+ testing a favorable option. Finally, ART at the time of conception appears to be associated with improved outcomes and reduced transmission. Within our cohort, the majority of women receiving early ART were initiated on ART earlier than 40 weeks prior to delivery, indicating that they were receiving treatment at the time of conception. Results from cohorts in South Africa, the UK and Ireland, and France all suggest that women who become pregnant on ART have lower transmission rates as compared to women who initiate therapy after becoming pregnant (0.7% vs. 5.7%, p = 0.01; 0.1% vs. 1.3%, p = 0.001; and 16% vs. 45%, p = 0.17, respectively) [13], [31], [32]. Our results support the findings in these studies and provide support for lifelong ART to reduce future transmissions, as well as highlight the need to test women prior to becoming pregnant.

However, an important note is that option B+ in Malawi uses an efavirenz (EFV)-based ART regimen, as compared to the NVP based treatment regimen used in our cohort. The potential risk of rare EFV related teratogenicity in the first trimester [33] needs ongoing monitoring, especially given the number of HIV-infected women who may have repeat pregnancies while on EFV. This potential risk also further highlights the need for family planning services to be included and prioritized with roll out of option B+.

Despite support for early ART initiation and option B+, the efficacy of this approach will be compromised unless efforts are made to promptly link newly identified women to ART services and to ensure identification, enrollment into care, and testing of exposed infants. Significant challenges to early identification of women and prompt ART initiation remain [34]–[36], and various studies have begun to identify some of these obstacles [37], such as economic concerns, stigma, obstruction from spouses or families, and suboptimal provider-patient interactions. The barriers to initiation of and adherence to ART [38] need to be investigated further so that effective interventions can be developed. Finally, there are early data from the routine monitoring systems in Malawi demonstrating a dramatic increase in the number of pregnant women initiating ART and retention at 6 and 12months similar to non-pregnant adults on ART [30]. However, additional studies to support these findings and further evaluate the impact of option B+ on HIV transmission, long-term retention, and outcomes are needed.

The most important limitation in our study was loss-to-follow-up (LTFU). Not all HIV-infected women identified at our clinics joined our program, and 14% of our enrolled cohort were lost or refused ongoing care after enrollment. These results compare favorably with other PMTCT programs, in which LTFU rates of 25–75% have been observed [12], [13], [18]. However, our transmission rates did not include those LTFU (lost, moved, refused care) and it is very possible that outcomes were worse in this group. Although we cannot confirm this, those LTFU likely did not receive a complete course of ARVs. Furthermore, the results from this study suggest that, compared to those with first PCR results available, those women who were LTFU were more likely to be younger in age and have newly diagnosed HIV; they were also less likely to be on ART at enrollment, and to have partners who were unaware of their HIV status. These characteristics may have also contributed to worse outcomes including increased vertical HIV transmission. CHW support of women to aid in the disclosure process as well as the creation of improved opportunities for antenatal couples testing might improve HIV disclosure to partners, thus leading to decreased maternal loss to follow-up and overall decreased MTCT. Innovative solutions to address reasons for LTFU and improve completion of the PMTCT cascade are needed to eradicate vertical transmission.

There were other limitations to our study. Since we included all first PCR test results and did not use a strict age range of 6–8 weeks, our findings are not directly comparable with other studies that use this age cut off. In Malawi, infant feeding guidelines recommend breastfeeding until children reach two years of age. The vast majority of our cohort continues to breastfeed, therefore, final infection status will be reported later.

These results provide reassurance that low HIV transmission rates can be achieved even in resource-limited settings. Maximum benefit is received from ART started at least 14 weeks prior to delivery for eligible women versus other regimens. Furthermore, more advanced HIV disease as indicated by lower baseline CD4+ cell count does not impact transmission among women in the setting of appropriate provision of maternal ART and PMTCT prophylaxis. Improving timely initiation of ART and PMTCT prophylaxis is essential to effectively reduce MTCT of HIV on a global scale.
